# Effects of the COVID-19 pandemic on elderly patients with head and neck squamous cell carcinoma

**DOI:** 10.3389/fonc.2022.966011

**Published:** 2022-09-23

**Authors:** Pei-Jing Ye, Yan Xi, Chuan-Zheng Sun, Qian Lei, Lei Li

**Affiliations:** Department of Head and Neck Surgery Section II, The Third Affiliated Hospital of Kunming Medical University, Kunming, China

**Keywords:** Head and neck squamous cell carcinoma (HNSCC), COVID-19, diagnosis delays, treatment interruptions, elderly patients

## Abstract

**Background:**

The 2019 novel coronavirus disease (COVID-19) strongly affects health care activities in countries around the world. The diagnosis and treatment of cancer have also been involved, and elderly head and neck squamous carcinoma is one of them. This study aimed to assess the impact of COVID-19 on elderly patients with head and neck squamous cell carcinoma (HNSCC) in our center.

**Methods:**

This retrospective study analyzed the clinical characteristics of 400 HNSCC patients over 65 years of age, calculated their treatment interruption rates, and compared the time of delayed diagnosis.

**Results:**

The rate of elderly patients with HNSCC with a delayed diagnosis was higher in the “during COVID-19 pandemic” group (DCOV19 group) than in the “during COVID-19 pandemic” group (BCOV19 group), and the difference was statistically significant (*p*=0.0017). There was a substantial difference in the rate of treatment interruption between the two groups (*p*=0.002).

**Conclusions:**

This is the first study to explore the effect of the COVID-19 pandemic on visits and treatment interruptions in elderly patients with HNSCC. The current impact of the COVID-19 pandemic on HNSCC treatment has resulted in reductions and delays in diagnosing cancer and providing treatment.

## Introduction

The 2019 novel coronavirus disease (COVID-19) was first observed in the Republic of China at the end of December 2019. Since the emergence of the COVID-19 pandemic, steps have been taken to mitigate the spread of the virus and have changed almost everyone’s lifestyles. Public health orders have established lockdowns in some regions and issued stay-at-home orders due to the highly infectious virus that is transmitted by respiratory droplets and contact. The prolonged pandemic has limited the movement of people within a community. In this environment, people have become conscious of physical distancing and putting on masks. The COVID-19 pandemic has posed pressing challenges in providing health care to cancer patients (1).Due to lockdowns, a large part of screening and diagnosis has had to be postponed (2, 3). During the pandemic, the cancer mortality rate was higher than that of the new coronary pneumonia (4), and an advanced clinical stage and treatment interruption were risk factors (5).

Approximately 30% of patients with head and neck squamous cell carcinoma (HNSCC) are at least 70 years old when they first seek medical care. The majority of oral cancer patients are between the ages of 50 and 80 years old. This number continues to grow as the population ages and life expectancy increases. During the COVID-19 pandemic, cancer management has been affected by various reasons, particularly for elderly patients with head and neck squamous cell carcinoma, who are considered high risk for infection with COVID-19. On the other hand, head and neck surgeons are also faced with unprecedented challenges and face not only increased exposure to infectious respiratory droplets and upper respiratory infection but also disruptions to treatment plans in screening, diagnosis, treatment, and rehabilitation due to the pandemic (6). Thus, the number of preventable cancer deaths is expected to increase because of delays in diagnosis during the COVID-19 pandemic (7). Recently, research has shown that an increase in cancer-related deaths would occur because of the effect of the pandemic on health systems. We hypothesized that delays in diagnosis and the interruption of treatment would lead to poor prognosis in elderly patients with HNSCC. According to international consensus, there was strong agreement that delaying treatment for too long is unacceptable (8). The COVID-19 pandemic has a certain impact on the diagnosis and treatment of the disease (9), but the impact on elderly patients with HNSCC is still unclear. Unfortunately, at present, there are few studies on the impact of elderly patients with HNSCC. Our research aimed to explore the effects of the COVID-19 pandemic on elderly patients with HNSCC. The research highlights the potential risk of treatment interruption and delays in diagnosis related to COVID-19 in elderly patients with head and neck cancer.

## Materials and methods

### Information and patient selection

This retrospective review included 400 patients with head and neck squamous cell carcinoma (HNSCC) presenting in our center. All consecutive patients with newly diagnosed head and neck squamous cell carcinoma (HNSCC) were seen between January 2011 and January 2022 in the outpatient clinics of the Third Affiliated Hospital of Kunming Medical University were included. This retrospective cohort study aimed to compare the impact of the COVID-19 pandemic on treatment interruption and delays in diagnosis in elderly patients with HNSCC. We divided these patients into the “before the COVID-19 pandemic” (BCOV19 group) and “during the COVID-19 pandemic” groups (DCOV19 group), defined as January 2011 to December 2019 and December 2019 to January 2022, respectively.

### Inclusion criteria and exclusion criteria

The following inclusion criteria applied: (1) Aged 65 years or older (calculated from the date of birth to the date of diagnosis of the case); (2) HNSCC diagnosed by cytology and/or histopathology (including oral cancer, oropharyngeal cancer, laryngeal cancer, hypopharyngeal cancer); (3) Follow-up time ≥ 1 month. The following exclusion criteria applied: At the same time combined with the second primary cancer.

### Statistical analysis and definition

The data were analyzed using SPSS 24. The chi-square test or Fisher’s exact test was used to compare categorical variables. A *p* value < 0.05 was considered statistically significant.

Cancer diagnosis delay: Individuals who had symptoms for more than 1 month and sought medical treatment belonged to the delayed group, which we further divided into two groups: delayed within 6 months and delayed for more than 6 months.

Treatment interruption was defined as the absence of treatment for greater than 3 weeks, including but not limited to radiotherapy and chemotherapy.

To assess whether the COVID-19 pandemic led to decreases in the ratio of patients who received surgery, we divided the treatment methods into surgical and nonsurgical treatments. We used the screening tool Geriatric 8 (G8) to assess elderly patients’ frailty (scores less than or equal to 14 were considered frail).

This study was authorized by the Ethics Committee of the No. 3 Affiliated Hospital of Kunming Medical University, with all patients providing consent.

## Results

### General characteristics

There were 400 elderly patients with HNSCC aged from 65 to 89 years in our center, with 340 (85%) men and 60 (15%) women. The clinical characteristics of elderly HNSCC patients with different periods are shown in **Table 1**, and the differences in the number of observed clinical features among elderly HNSCC patients with different sublocations were compared. Of these patients, 126 (31.5%) had oral cancer, 102 (25.5%) had laryngeal cancer, 78 (19.5%) had oropharyngeal cancer, and 94 (23.5%) had hypopharyngeal cancer. All patients accepted regular antitumor treatment, including surgical treatment (n=104, 26%) and nonsurgical treatment (n=296, 74%). Seventy-eight (23.2%) of BCOV19 group required nutritional support, while 20(31.2%) of DCOV19 group required nutritional support. There is no statistically significant difference between the groups (*p >*0.05). One-hundred and fifty-two (45.2%) of BCOV19 group ECOG score is 0-1, while 44 (68.8%) of DCOV19 group with the ECOG score 0-1. The difference was statistically significant. (*p*=0.001) In BCOV19 group, 159(47.3%)were assessed as frail (G8), while 21(32.8%) in DCOV19 group, with statistically significant difference (*p*=0.045) (**Table 1**).

**Table 1:** Clinical characteristics of the 400 elderly patients with head and neck cancers.

Clinical characteristics	BCOV19	DCOV19	P
Age (yrs.)			0.945
65≤Age<70	162 (48.2%)	30 (46.9%)	
70≤Age<75	98 (29.2%)	20 (31.3%)	
Age≥75	76 (22.6%)	14 (21.8%)	
Sex			0.359
Male	288 (85.7%)	52 (81.3%)	
Female	48 (14.3%)	12 (18.7%)	
Tobacco Use			0.637
Yes	210 (62.5%)	38 (59.4%)	
No	126 (37.5%)	26 (40.6%)	
Smoking Index			0.346
<800	100 (29.8%)	14 (21.9%)	
≥800	122 (70.2%)	24 (78.1%)	
Alcohol Use			0.479
Yes	142 (42.3%)	24 (37.5%)	
No	194 (57.7%)	40 (62.5%)	
BMI (kg/m^2^)			0.341
≤18.4	86 (25.6%)	20 (31.2%)	
18.5~23.9	184 (54.8%)	36 (56.3%)	
≥24.0	66 (19.6%)	8 (12.5%)	
ECOG			0.001
0~1	152 (45.2%)	44 (68.8%)	
≥2	184 (54.8%)	20 (31.2%)	
Comorbidities			0.106
Yes	152 (45.2%)	36 (56.3%)	
No	184 (54.8%)	28 (43.7%)	
aCCI			0.512
> 8	122 (36.3%)	26 (40.6%)	
≤8	214 (63.7%)	38 (59.4%)	
Frailty screeners			0.045
Frail (G8)	159 (47.3%)	21 (32.8%)	
Non-frail (G8)	177 (52.7%)	43 (67.2%)	
TNM Stage			0.721
Early(I~II)	102 (30.4%)	18 (28.1%)	
Advanced(III~IV)	234 (69.6%)	46 (71.9%)	
Sublocations			0.073
Oral cancer	98 (29.2%)	28 (43.8%)	
Laryngeal cancer	90 (26.8%)	12 (18.7%)	
Oropharyngeal cancer	70 (20.8%)	8 (12.5%)	
Hypopharyngeal cancer	78 (23.2%)	16 (25.0%)	

### Delayed diagnosis and treatment interruption

We divided 400 elderly patients with HNC into BCOV19 group and DCOV19 group. Our data showed a significant decrease in the number of newly diagnosed patients during the COVID-19 pandemic. Compared with BCOV19 group, there was no significant difference in the proportion of patients aged 75 years or older in DCOV19 group (**Table 1**).

Of these patients, 24 (37.5%) patients in DCOV19 group underwent surgery. However, 80 (23.8%) patients in BCOV19 group opted for surgical treatment. In BCOV19 group, 234 (69.6%) patients had an advanced TNM stage (III~IV), while in DCOV19 group, 46 (71.9%) patients had an advanced TNM stage. Differences in the tumor TNM stages between the groups were not statistically significant (*p >*0.05).

The rate of patients with a delayed diagnosis (53.1%) was higher in DCOV19 group than in BCOV19 group (32.7%). There was a significant difference in the rate of patients with treatment interruption between the two groups (*p*=0.002).

Twenty (31.2%) patients required nutritional support in DCOV19 group, while 78 (23.2%) patients in BCOV19 group required nutritional support, with no statistically significant difference (*p >*0.05) (**Table 2**).

**Table 2:** Comparison of clinical characteristics in the two groups.

	BCOV19 group	DCOV19 group	P
Delayed Diagnosis			0.002
Yes	110 (32.7%)	34 (53.1%)	
No	226 (67.3%)	30 (46.9%)	
Treatment Interruption			0.040
Yes	179 (53.3%)	43 (67.2%)	
No	157 (46.7%)	21 (32.8%)	
Nutritional Support			0.171
Yes	78 (23.2%)	20 (31.2%)	
No	258 (76.8%)	44 (68.8%)	
Surgery			0.022
Yes	80 (23.8%)	24 (37.5%)	
No	256 (76.2%)	40 (62.5%)	

### Delayed diagnosis

**Figure 1:**
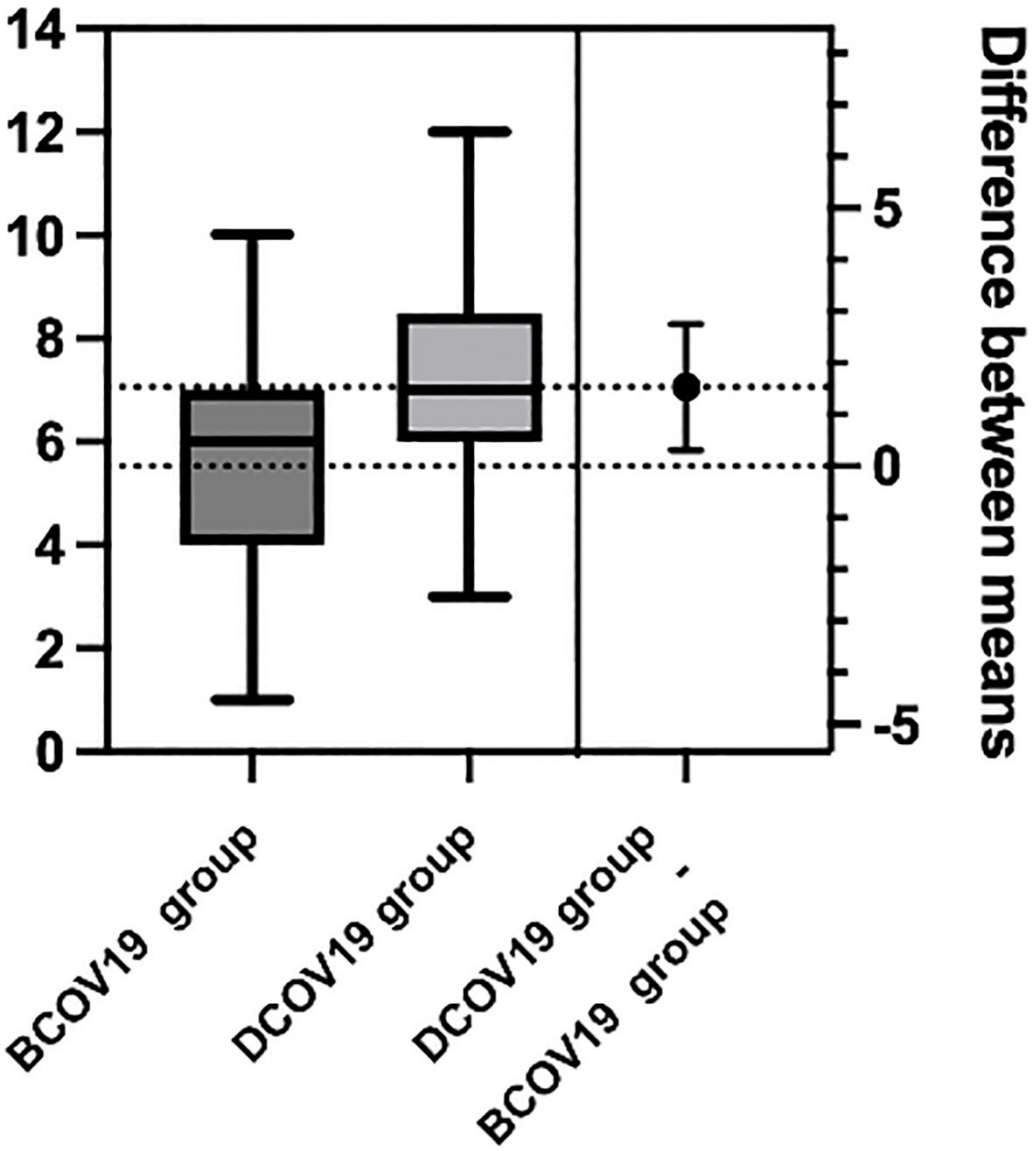
Comparison of median delayed diagnosis time in BCOV19 and DCOV19 groups.

Patients in BCOV19 group delays ranged from 1 month to 10 months, and the median is 5.5 months. Patients in DCOV19 group delays ranged from 3 months to 12 months, and the median is 7.0 months. So, we divided delayed diagnosis into within 6 months and more than 6 months two groups (**Table 3**). There were 66 (19.6%) patients who delayed diagnosis for more than 6 months in BCOV19 group compared with 28 (43.8%) patients in DCOV19 group. The median delayed time was 5.5 months and 7.0 months, respectively. A significant statistical difference was determined in the stage comparison (*p*= 0.0017) (**Figure 1**).

**Table 3:** Differences in the delayed diagnosis time between the two groups.

	BCOV19 group	DCOV19 group	P
Delayed diagnosis time			0.0017
Delayed ≤ 6 months	44 (13.1%)	6 (9.4%)	
Delayed > 6 months	66 (19.6%)	28 (43.8%)	
Total	110 (32.7%)	34 (53.2%)	

### Treatment interruption

Among the included patients, 179 (53.3%) patients in BCOV19 group experienced treatment interruption, compared with 43 (67.2%) patients in DCOV19 group. There was a significant difference between BCOV19 group and DCOV19 group (*p*=0.040).

## Discussion

This retrospective single-institution study included 400 elderly patients with HNSCC.

These patients were seen at our institution from January 2011 to January 2022 to investigate the impact of the COVID-19 pandemic on diagnosis delays and treatment interruptions. According to the World Health Organization statement, we defined BCOV19 group and DCOV19 group as of January 2011 to December 2019 and December 2019 to January 2022, respectively (10). COVID-19 presents unprecedented challenges in diagnosing and treating elderly patients with HNSCC. In underdeveloped areas in China, it is not easy for most elderly patients to seek medical treatment alone. Most elderly patients need to be accompanied by their family members, especially those living in rural areas. This situation has become more serious during the COVID-19 pandemic. This is the first study to explore the effects of the COVID-19 pandemic on visits and treatment interruptions in elderly patients with head and neck squamous cell carcinoma. Our research shows that rates of diagnosis delays and treatment interruption in elderly patients with HNSCC have increased between 2020 and 2021 in our institution. There was a modest reduction in the number of elderly patients diagnosed with HNSCC between December 2019 and January 2022 compared to the number of patients diagnosed in 2019. Multiple studies have shown that cancer patients are less likely to visit hospitals globally during the COVID-19 pandemic (11, 12). Maring et al. estimated that the number of people seeking health care decreased and that accessibility, as well as the availability of diagnostic services, declined during the COVID-19 pandemic (13). This has led to a decrease in newly diagnosed cancers worldwide (14). This may be related to the various restrictions imposed in the context of a public health emergency. Additionally, this may be due to a patient’s fear of the pandemic or other factors (15). Undoubtedly, more aggressive regulation can help control the spread of the virus. Nevertheless, it also poses new challenges for elderly cancer patients.

In geriatric oncology, frailty status is an emerging indicator with prognostic value. The incidence of frailty is exceptionally high in elderly cancer patients, as both cancer and the therapies provided can be critical additional stressors that challenge the patient’s physiological reserves (16). Among them, elderly patients with head and neck squamous cell carcinoma have more frailty than patients with other solid malignancies (17). Therefore, identification and active management of frailty will help cancer patients better tolerate cancer treatment (18). In our study, in BCOV19 group, 159(47.3%) were assessed as frail (G8), while 21(32.8%) in DCOV19 group, with a statistically significant difference (*p*=0.045). To a certain extent, the ECOG score reflects the patient’s frail status. In our research, the ECOG score has the same trend as frailty (G8). One-hundred and fifty-two (45.2%) of BCOV19 group’s ECOG score is 0-1, while 44 (68.8%) of DCOV19 group with the ECOG score of 0-1. The difference was statistically significant. (*p*=0.001) This means that the basic condition of these patients is relatively good. The general condition of the patients in DCOV19 group was better than BCOV19 group at the consultation time. This may be because the general condition of patients who can come to the hospital during the epidemic is better. We found that, compared with that of elderly HNSCC patients before the pandemic, the admission delay rate of elderly HNC patients in our center significantly increased during the pandemic. In addition, there was also a significant difference in the treatment interruption rate of BCOV19 group and DCOV19 group. We also found that more elderly patients with HNSCC required nutritional support upon admission. Furthermore, the public health order suggested staying at home and avoiding close contact, restricting patient access to medical services and increasing this situation. Moreover, the COVID-19 pandemic and the associated community mitigation efforts have altered health care delivery and access to health care. Fewer older patients are being screened during the pandemic, resulting in a decreasing number of cancer diagnoses, possibly due to patient fears of the COVID-19 pandemic (6). Lazzerini et al. found that the number of pediatric emergency patients significantly decreased during the lockdown of the COVID-19 pandemic in Italy, which may have been due to the reallocation of medical resources during the lockdown and parents’ fears of contacting COVID-19 during medical treatment (11). Children and elderly individuals are two special groups in society; both have a higher risk of severe illness and more complications due to a lack of access to medical care. Our research shows that although delays in diagnosis and interruptions in treatment are common in elderly patients with HNSCC, this phenomenon appears to be more pronounced during the pandemic. Overall, the number of avoidable cancer deaths is expected to rise significantly due to delays in diagnosis during the COVID-19 pandemic.

A predictable consequence of cancer screening and diagnosis delays is that cancer will appear at an advanced stage, which often requires more complex management. We anticipate that the impact of the COVID-19 pandemic on access to cancer treatment will lead to stage migration to more advanced disease stages and an overall increase in cancer mortality. Tevetoglu et al. reported that during the COVID-19 pandemic, the number of T3- and T4-stage head and neck cancers increased significantly compared with the number before the COVID-19 pandemic (4). Our study found that the TNM stage of elderly patients with HNSCC was more advanced during the COVID-19 pandemic than before the COVID-19 pandemic, but this was not statistically significant. This may be because, as our hospital is the only oncology hospital in Yunnan Province, most patients are at an advanced stage when they come to our hospital.

Several studies have shown a decline in the number of surgeries during the pandemic. Our institution also recorded an overall decrease in clinical and surgical activity during 2020. Yigit et al. discovered that one of the most significant variations in the health care environment during the COVID-19 pandemic had been in surgical management strategies. Foremost among these are the restrictions of elective surgical procedures and the priority of emergency or nondelayed tumor surgery (19). In Germany, during the COVID-19 pandemic, the number of hepatic-pancreato-biliary surgeries and oncologic surgeries decreased to varying degrees (20, 21). During the pandemic. During the pandemic, we have seen an increase in elderly patients with HNSCC presenting to our center who choose surgery as a treatment modality. This may be because while seeking medical care is more difficult during the pandemic, the willingness of patients to seek medical care is more vital than before the COVID-19 pandemic. Schoonbeek et al. found that delayed treatment initiation was independently associated with increased recurrence risk in patients treated with initial surgery (22). According to treatment delays were not significantly associated with overall survival or tumor recurrence in cancer patients (23). But this also prompts us to attach great importance to the early treatment and timely diagnosis of elderly patients with HNSCC.

Although this retrospective study confirmed the impact of delayed diagnosis and treatment interruption on elderly HNSCC patients, there has been no specific study on the particular causes of diagnosis delays and treatment interruptions. It was impossible to solve patients’ difficulties in seeking medical treatment for detailed reasons. Under the new situation, we should consider how to formulate screening, diagnosis, and treatment strategies for elderly patients with head and neck squamous cell carcinoma because the current global COVID-19 trend shows that it may last much longer than expected and is more likely to become endemic after the pandemic.

## Conclusion

The current impact of the COVID-19 pandemic on the elderly patients with HNSCC treatment has resulted in reductions and delays in diagnosing cancer and providing treatment. This problem may increase head and neck cancer morbidity and mortality in the coming years.

## Data availability statement

The data that support the findings of this study are available from the corresponding author upon reasonable request.

## Author contributions

Conception and design: LL and P-JY. Collection and assembly of data: LL, P-JY, and QL. Data analysis and interpretation: C-ZS and YX. Manuscript writing: LL and P-JY. Final approval of manuscript: All authors.

## Funding

The present study was supported by the National Natural Science Foundation of China (Grant No.82060499).

## Conflict of interest

The authors declare that the research was conducted in the absence of any commercial or financial relationships that could be construed as a potential conflict of interest.

## Publisher’s note

All claims expressed in this article are solely those of the authors and do not necessarily represent those of their affiliated organizations, or those of the publisher, the editors and the reviewers. Any product that may be evaluated in this article, or claim that may be made by its manufacturer, is not guaranteed or endorsed by the publisher.

## Abbreviations

HNSCC, Head and Neck Squamous Cell Carcinoma; BCOV19 group, The “before COVID-19 pandemic” group; DCOV19 group, The “during COVID-19 pandemic” group; aCCI, Age-adjusted Charlson Comorbidity; BMI, Body Mass Index; ECOG, Eastern Cooperative Oncology Group.
